# Draft Genome Sequence of a European Isolate of the Apple Canker Pathogen *Neonectria ditissima*

**DOI:** 10.1128/genomeA.01243-15

**Published:** 2015-11-19

**Authors:** Antonio Gómez-Cortecero, Richard J. Harrison, Andrew D. Armitage

**Affiliations:** East Malling Research, East Malling, Kent, United Kingdom

## Abstract

The *Sordariomycetes* fungus *Neonectria ditissima* is a major pathogen of apples, causing canker on trees and fruit spoilage. We report here the draft genome sequence of a European strain isolated from cankerous tissue.

## GENOME ANNOUNCEMENT

European canker caused by the phytopathogenic fungus *Neonectria ditissima* is a severe economic problem for apple growers, particularly in northwestern Europe and temperate regions (1, 2). This pathogen causes yield loss through the direct infection of wood and pre- and postharvest rot of apple fruits (3). Conidia and ascospores can initiate infection during all growth stages (4), meaning successful control by fungicide application and good horticultural practice is difficult to achieve (5, 6). Pyramiding resistance genes into new cultivars offers a promising strategy for control, as differential resistance has been observed between apple cultivars (2, 7). However, the success of breeding programs requires foresight, as the breeding cycle of the apple is slow. Generating the genome of *N. ditissima* provides resources for effector identification. This will aid research into the basis and durability of resistance.

Isolate R09/05 was obtained in 2005 from a canker-infected wood of *Malus domestica* cv. “Cox” in Kent, United Kingdom. A single ascospore culture of R09/05 was used for the genome sequencing. DNA extraction was performed on freeze-dried mycelium using a GenElute plant DNA miniprep kit (Sigma-Aldrich). The manufacturer’s protocol was modified by doubling the volume of lysis solutions used, performing an RNase digestion step, and using twice the volume of precipitation solution. Paired-end genomic libraries were prepared using a Nextera sample preparation kit (Illumina), according to the manufacturer’s protocol. Analysis using a fragment analyzer (Advanced Analytical Technologies) confirmed that libraries had a high representation of DNA fragments 600 to 1,000 bp in length. Libraries were sequenced using 300-bp reads on an Illumina MiSeq machine, resulting in 5,389,629 paired-end reads.

Adaptor sequences and low-quality data were removed using fastqc-mcf. The sequencing depth was estimated to be 42× and genome size to be 48 Mb, following *k*-mer counting using KMC (8). *De novo* assembly was performed using SPAdes version 3.1.0 and analyzed using Quast (9). The genome was assembled into 45.72 Mb in 675 contigs (>500 bp and >10× coverage), with an *N*_50_ metric of 154 kb, a largest scaffold of 688 kb, and a G+C content of 50.84%. RepeatModeler and RepeatMasker were used to identify repetitive and low-complexity regions within the assembly, masking 11.67% (5.33 Mb) of the genome (http://www.repeatmasker.org). Cegma (10) was used to assess gene space, identifying that 236 of 248 (95.16%) core eukaryotic genes were present in the assembly. Gene prediction was carried out using AUGUSTUS 3.1 (11), which predicted 12,711 genes from the unmasked assembly. A draft functional annotation was determined for the gene models using InterProScan-5.8-49.0 and through identifying homologies between predicted proteins and those contained in the Swiss-Prot database (September 2015 release) using BlastP.

### Nucleotide sequence accession numbers.

This whole-genome shotgun sequencing project has been deposited at GenBank under the accession no. LKCW00000000 (BioProject PRJNA292426). The version described in this paper is the first version, LKCW01000000.
